# Lay perceptions of risk factors for Rift Valley fever in a pastoral community in northeastern Kenya

**DOI:** 10.1186/s12889-016-2707-8

**Published:** 2016-01-13

**Authors:** Caroline M. Ng’ang’a, Salome A. Bukachi, Bernard K. Bett

**Affiliations:** 1 Institute of Anthropology, Gender and African Studies, University of Nairobi, Nairobi, Kenya; 2 International Livestock Research Institute, Nairobi, Kenya

**Keywords:** Lay perceptions, Food consumption practices, Rift Valley fever, Risk factors, Pastoralism

## Abstract

**Background:**

Human behavioral factors have been found to be central in the transmission of Rift Valley fever. Consumption of contaminated meat and milk in particular have been identified as one of the key risk factors for the transmission of Rift Valley fever in humans. In pastoral communities, livestock is the main source of livelihood from which many benefits such as food as well as economic and cultural services are derived. Zoonotic diseases therefore have a great impact on pastoral communities livelihoods. However, lay perceptions regarding the transmission of these diseases including Rift Valley fever hampers their effective control. This study investigated the lay perceptions of risks for Rift Valley fever transmission in a pastoral community in northeastern Kenya.

**Methods:**

A qualitative study was carried out in Ijara district, Kenya which was one of the hotspots of Rift Valley during the 2006/2007 outbreak. Data were collected using focus group discussions and narratives guided by checklists. Eight focus group discussions consisting of 83 participants and six narratives were conducted. Data was transcribed, coded and analysed according to Emergent themes.

**Results:**

The participants reported that they had experienced Rift Valley fever in their livestock especially sheep and in humans both in 1997/1998 and 2006/2007. However, they believed that infections in humans occurred as a result of mosquito bites and had little to do with their consumption of meat, milk and blood from infected livestock. The participants in this study indicated that they had heard of the risks of acquiring the disease through consumption of livestock products but their experiences did not tally with the information they had received hence to them, Rift Valley fever was not transmissible through their dietary practices.

**Conclusions:**

Though the communities in this region were aware of Rift Valley fever, they did not have elaborate information regarding the disease transmission dynamics to humans. To avoid misconception about transmission of the disease, intervention strategies, require to be accompanied by comprehensive explanations of the dynamics of its transmission. It is necessary to develop appropriate interventions that take into consideration, lay perceptions of risk factors for the disease and communities’ livelihood strategies.

## Background

Rift Valley Fever (RVF) is a zoonotic disease first recognized and characterized in the Rift Valley region of Kenya in 1931 [1–3]. It affects animals such as cattle, sheep, camels, goats as well as humans [4, 5] and can be transmitted between humans and animals [6]. Humans acquire RVF infection from bites of infected mosquitoes, exposure to the blood, body fluids and tissues of infected animals as well as inhaling infectious aerosols from body tissues [2]. Most infections in humans are asymptomatic and therefore result in no symptoms or in mild illness [5]. However, a significant number of RVF infected patients develop severe disease which includes hemorrhage, encephalitis, visual disturbances and death [2]. Additionally, studies have shown that RVF is affecting humans more because of its highly virulent form thus causing increasingly high fatality rates in subsequent outbreaks [7]. The morbidity and mortality from RVF on both humans and animals leads to low productivity, the diversion of often limited household resources to address ill health and loss of income through slaughter bans and quarantines as these populations often solely depend on livestock for their sustenance [5, 7–9]. Therefore, the losses caused by the RVF outbreaks continue to play a role in perpetuating poverty and further compromising attempts to improve the well-being of the world’s poorest people [9–11].

In Kenya there have been outbreaks most recently in 1997–1998 and 2006–2007 [2, 4, 5]. These outbreaks mainly occurred in the Garissa and Ijara regions, which are predominantly pastoral communities [12]. These are areas characterized by seasonal vector activity as a result of periodic heavy rainfall and the subsequent flooding [1, 13]. Consequently, these vectors are responsible for the transmission of RVF in these regions [1, 13]. Human behavior has also been implicated in the transmission and spread of RVF [2, 4, 5, 12, 14]. This relates to several activities associated with human- animal exposure such as contact with the blood, secretions, tissues or body fluids of infected animals during slaughter, food preparation, assisting with animal births or conducting veterinary procedures [12, 15].

The low awareness and poor knowledge on the relationship between zoonoses, food consumption practices and animal husbandry practices have been identified as likely to expose pastoral communities to a high risk of contracting zoonoses due to their consistent close interaction with livestock [16]. Pastoral communities in general depend primarily on livestock for their livelihoods, nutrition, companionship and socio economic development [4, 17, 18, 19]. It has been established that up to 90 % of the population in the Ijara region are dependent on livestock for food and income [4, 18]. In regard to food consumption practices, consumption of raw blood mixed with milk or hot soup is common while the boiling of milk is uncommon in pastoral communities [15].

Lay perceptions regarding diseases are important because preventive practices related to any disease require the adherence of the population in question to these practices [19] yet most studies on RVF [2, 4, 5, 12, 15] have only focused on the risk factors associated with RVF but few have sought to understand how the local communities conceptualize those risk factors. According to the Health Belief Model (HBM), people take certain health related actions only if they believe that action will prevent a particular disease [20]. The HBM is relevant in understanding the perceived threat from disease, understanding lay beliefs and how these beliefs influence health behaviour and the adoption of preventive practices [21]. Thus beliefs are important in inducing prevention related behavior to a community [22]. For example adherence to any preventive and control strategies for any disease by an individual are less effective when that individual’s attribution for disease differs from the patho-physiological causes of that disease [23, 24]. Similarly, if a community’s causal explanations for disease differ from those by public health officials there is a greater chance of lack of adherence to any preventive practices [25]. Indeed, in the case of RVF the main constraint for the control and prevention of RVF has been identified as inadequate knowledge by the communities of the risk factors involved in the disease’s occurrence and maintenance [26]. A better understanding of lay perceptions and underlying assumptions about disease risk may be useful in discussing disease risk and risk reduction strategies with communities. This work was done to describe the lay perceptions of the local communities regarding the risk factors for RVF and food consumption practices.

## Methods

### Study site and population

**Fig. 1:**
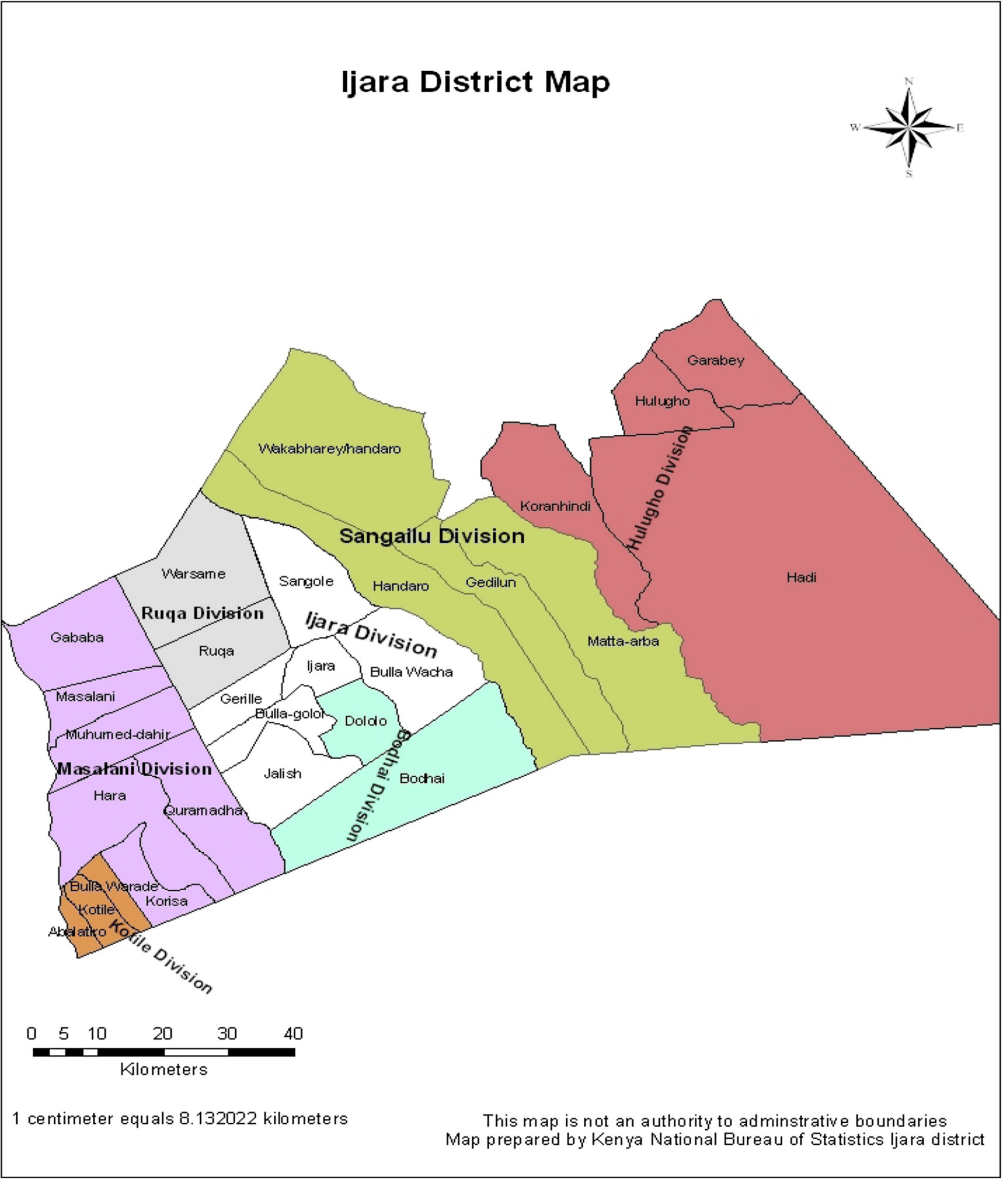
Map of Ijara District (Source KNBS, 2000)

This study was carried out in Ijara Division of Ijara District in North Eastern Province (Fig. 1). The district borders Fafi District to the North, Lamu District to the South, Tana Delta District to the South West, Tana River to the West and the Republic of Somalia to the East. This study was conducted in the Ijara and Bulla Golol locations of Ijara Division. The two locations are approximately 50 km apart. Ijara Division has a total population of 19,259 people [27]. These areas were chosen because they were one of the regions where the RVF outbreaks have consistently occurred and at a great magnitude [5, 12]. Majority of the population in this area are ethnically Somali pastoralists. Generally, the county is sparsely populated with majority of the population being concentrated in facility and service areas. The population depends on pastoralism for livelihood and has great value for animals.

The study population consisted of the adults in Ijara district in Garissa County. The participants were aged 18 years and above both males and females. Focus group discussions and narratives were used to collect qualitative data.

### Data collection

This research was conducted between August 2013 and October 2013. This study was part of a larger study titled, the Dynamic Drivers of Disease in Africa. Other aspects of RVF that were investigated in the larger study included land use and animal husbandry practices, herd management, environmental and livelihood changes over the past 50 years and determining the environmental- health context through participatory mapping, interviewing and ranking exercises.

#### Focus group discussions

Community members were recruited to participate in the focus group discussions through local administrators and elders. People were selected to participate if they were adults (18 years and above), had resided in the community for not less than 15 years and had a range of experiences with RVF. Additionally men and women were recruited to be interviewed separately to avoid dominance by either gender. The people were recruited from a wide age range (18–60 years) to provide a wider scope of information. Using a discussion guide, four focus group discussions (FGDs) were conducted for each gender with each group having between 9–12 participants.

The FGDs participants were asked open ended questions about types of livestock kept, uses of livestock, benefits of livestock, causes of RVF, signs and symptoms of RVF and perceived risk factors for RVF that relate to their food consumption practices. Participatory mapping and ranking exercises were used to understand the community’s perceptions of RVF causality. The FGDS were held in the village either in the morning or in the afternoon under a tree or shade for between 60 to 75 min. Local male elders were also present during the interviews to clarify issues once the discussion was over. The discussions were facilitated by a moderator, a note taker and an interpreter who was fluent in the local language (Somali), national language (Kiswahili) and English. All the FGDs were recorded using digital recorders.

#### Narratives

Following the conclusion of the FGDs, a total of six narratives were conducted with informants who had an immediate family member infected with RVF (5) or had been infected in the last outbreak with RVF (1). Narratives were used to obtain a detailed profile of the personal lived experience with RVF regarding the symptoms, belief on causation, food consumption practices and the health seeking behavior. These narratives were conducted in a location suitable to the interviewee such as their home or in a quiet location within the local shopping centre. Each interview took place within 60 to 90 min.

Open ended questions were asked regarding the symptoms of RVF in humans and animals and the perceived risk factors for RVF. In addition, a description of their personal experience with RVF was sought such as when they got ill, where they were when they got ill, what they were involved in around that time, what they felt caused their illness, how long they were ill, extent of illness and the actions taken, experience with the treatment be it local or biomedical and the impact the illness had on them and their families. In each interview, notes were taken and the interview was also recorded. An interpreter was also present in all the narrative interviews.

### Data analysis

The recorded data were transcribed and checked against the notes that were taken during the interviews to ensure consistency. Content analysis was undertaken based on the research objectives and recurring themes, similar patterns and supportive quotations [28]. This involved reading and re-reading through the transcripts to familiarize with the data, manually generating initial codes, collating codes into identified recurring themes and analyzing manually using the refined themes and relating to research objectives.

#### Ethics statement

This study was part of a larger study titled Dynamic drivers of diseases in Africa carried out by the International Livestock Research Institute, Kenya. The ethical clearance was issued by the Ethical Review Committee of the African Medical Research Foundation Reference number AMREF-ESRC P65/2013. The research permit was obtained from the National Council of Science and Technology in the Ministry of Higher Education, Science and Technology. Ethical considerations were observed throughout the study. Verbal and written consent for participation in the study was sought from all the adults recruited after they were given information about the study. Only those who consented in writing to participate in the study were interviewed. To ensure anonymity and confidentiality of the participants, personal identifiers were removed in the final report. Immediately after each discussion or narrative interview, clarifications on issues raised by the discussants and informants were made. Some of the clarifications involved providing information on causes, symptoms and treatment of RVF. One feedback session at the community level was organized by the bigger project to disseminate preliminary findings from the study but more feedback sessions have been planned to take place at the end of the project.

## Results and discussion

The eight FGDs consisted of 44 women and 41 men in total while four of the informants for the narrative were men and two were women (Table 1).

**Table 1:** Number of participants in the study per category

Method	Category	Number
Focus Group Discussions	Women (Group 1)	11
	Women (Group 2)	10
	Women (Group 3)	10
	Women (Group 4)	11
	Men (Group 1)	9
	Men (Group 2)	10
	Men (Group 3)	11
	Men (Group 4)	11
	TOTAL	83
Narratives	Male	4
	Female	2
	TOTAL	6
Total of participants in the study		89

### The value of livestock in the community

The participants in this study (8/8 FGDs) reported that they kept various livestock such as cattle, goats, sheep, donkeys and chicken as their main source of livelihood. The reported benefits derived from livestock included food (meat, milk), hides and skin and income from their sale. Other cultural benefits cited were sheep fat which was used for cooking, treating pneumonia, stomach discomfort, typhoid and wounds. Raw blood was also useful as it was consumed by women to replenish blood lost during delivery and to cure stomach ailments. Consumption of sheep urine was also cited as a benefit to treat “Yellow fever”. Payment of dowry was reported to be carried out primarily using livestock while goats and sheep were normally slaughtered during community celebrations and for visitors.

Gender differences were reported in the proportions of livestock kept by species. Men reported that the community mainly kept goats followed by cattle, sheep, chicken and donkeys in that order (4/8 FGDs). Women on the other hand noted that the community mainly kept sheep followed by goats, cattle, chicken and donkeys in descending order (4/8 FGDs). The men gave the reasons they preferred to keep more goats as: being drought resistant; fetching better market prices; the meat tasting good; producing more milk than sheep and easier to milk than sheep. On the other hand, women reported that sheep were more preferred because: they produced a lot of fat when slaughtered; their fat was useful for cooking and as food for nursing mothers while the raw blood from sheep was useful in replenishing blood lost during child birth. Sheep, according to the women were also cheaper to purchase; reproduced faster within 5 months; its milk was tastier in tea and was also on high demand in the market.

Additionally, in all the FGDs, there was consensus that raw blood, animal fat and raw milk from livestock were used to treat people suspected to be suffering from RVF as exemplified in the excerpt below:

*I was herding cattle at the time when I got sick. I had a headache*…. *I was afraid of going to hospital. So I consumed raw blood from a goat as well as animal fat and milk mixed with water so that I could diarrhea and recover. However*, *I did not recover and I had to go to a health facility*. ….” **Narrative 3**: **49 year old male who was infected with RVF and survived**

The preceding narrative illustrates the perceived medicinal value attached to livestock products. Other studies have shown that fat from sheep was consumed to treat illnesses including patients manifesting RVF symptoms such as fever and bloody diarrhea [18]. Products from sheep are highly valued, especially by women, due to their perceived medicinal and dietary value yet sheep were reported to be the most affected species of livestock by RVF [2, 12, 15]. This may have important implications on the gender dynamics of RVF infections. This notwithstanding, much as previous studies have indicated that men and women are likely to be differentially exposed to RVF infection depending on the roles traditionally ascribed to them, males have been reported to be three times more likely to be seropositive than females because their main role as herders cause then to interact closely and for longer periods in isolation with animals hence increased vulnerability to RVF [18]. However, the manner in which male–female disaggregated roles among pastoralist communities differentially exposes women and men to infection and spread of RVF virus is not yet fully understood [18].

The value attached to livestock and the services that the community derives from them has implications on the transmission of RVF from animals to humans in case of an outbreak.

Livestock being a central part of the pastoralists’ lives and livelihood necessitates them to engage in certain practices however risky. These include residing with the animals in their houses at night in order to protect them from the floods, rain and cold and animals of prey. This has been identified as one of the risk factors for RVF [2, 5, 12, 29]. Furthermore, [2, 12, 14] noted that the enforcement of control strategies such as slaughter bans and bans on movement of livestock was difficult to implement due to the crucial role that livestock play in the lives of the communities in most of the epizootic prone areas. The design and implementation of interventions should take into consideration the role that livestock play in pastoral communities.

### Lay perceptions in relation to livestock related risk factors for RVF

The discussants perceived that RVF occurred as a result of mosquito bites during periods of unusually heavy rainfall and was not related to their food consumption practices (8/8 FGDs) as exemplified in the following quotes:

“*RVF is a dangerous disease and it is caused by mosquitoes. This occurs during periods of heavy rainfall and flooding like it did in 1997 and 2007*….” ***Men FGD1***, ***Ijara***.

“*RVF occurs when it rains heavily. This is because mosquitoes increase in number as a result of the long grass that grows. In addition*, *there is no wind to drive away the mosquitoes*”. ***Women FGD4***, ***Ijara***.

It was only after further probing that the participants in this study used words such as “the government said”, “we heard”, “people said” to describe RVF risk factors related to their food consumption practices. They mentioned (4/8 FGDs) that they had heard, mainly from Government agencies that consumption of meat and milk from diseased animals was a significant risk factor for RVF as well as other related exposures such as slaughtering, skinning and milking. Nevertheless they did not perceive this to be true as participants in all the FGDs reported to have consumed meat, unboiled milk and raw blood from their livestock during the last two RVF outbreaks. However, on further probing why they continued eating livestock products despite the government warnings, they attributed this to the lack of alternative sources of food, the need to salvage the meat from their livestock and the belief that God would protect them. This is illustrated in the following excerpt from a narrative:

“…*the Government said that consuming meat and milk also causes the disease. However*, *I personally believe it is mosquitoes that cause RVF and not meat. There were warnings through the radio against meat and milk consumption but we went ahead and consumed them and nobody got sick. We were eating the meat because the goats and sheep were dying very fast and they were very healthy so we slaughtered and ate. We just believed God would protect us*”. *And we also drank the milk*.” ***Male 35 years old***, ***Ijara***.

The cultural practice of slaughtering ill animals and cooking the meat was believed to make it safe to eat (8/8 FDGs) as illustrated below:

“*In our culture we believe that once meat has been boiled it has no disease and so it is fit for our consumption*”. ***Male 60 years old***, ***Ijara***.

The participants reported that according to their tradition, they do not butcher dead animals but they are allowed to slaughter very ill animals to salvage the meat as exemplified by a key informant in the following quote:

“*Doctors said we should not eat meat. We the Somalis believe that when our livestock get sick we should slaughter them and eat before they die*”. ***Male***, ***55 years old***, ***Ijara***.

In addition, sheep were the most affected livestock according to the community (8/8 FGDs) and they were dying in large numbers yet in all the women’s FGDs, the discussants reported that they consumed the meat from the sick sheep since they like the taste of mutton.

In spite of being in receipt of information that consumption of unboiled milk would expose consumers to RVF (5/8 FGDs), this practice was said to be common especially among women who normally consume raw milk while milking as well as share it with their young children to pacify them. Raw milk was preferred over boiled milk because it was perceived to taste good in spite of the risks involved. Women in one focus group discussion reported that, “*we do not wait to boil milk* (*laughter*). *When we are milking we taste some. We believe boiled milk doesn*’*t taste as good*”. ***Women FGD2***, ***Ijara***

In summary, the perceived consensus among all the discussants in the FGDs was that many of those that got infected with RVF were exposed to mosquito bites, for example, by sleeping outside without a mosquito net, and not as a result of consuming livestock products.

### Community knowledge gaps

This study identified three lay perceptions common about RVF transmission that may act as a barrier to the adoption of protective interventions or control strategies during RVF outbreaks. 1. It is only mosquitoes that cause RVF in livestock and humans through their bites especially during floods. Similarly, in their study in the Ijara region of Kenya, [26] found that the most important risk factors for RVF disease in humans as noted by the community were the high number of mosquitoes as well as high rainfall. Participants in that study observed that bites from infected mosquitoes at livestock watering points, around the cattle sheds and in bushy environments were considered a high risk pathway for acquiring RVF [26]. However, in Sudan, [30] stated that while mosquitoes played a role in the transmission of RVF to humans one of the most significant risk factors for severe RVF disease was consuming or handling products from sick animals. Studies conducted in Kenya too, demonstrated that the most significant risk factors for RVF were slaughter as well as consumption of meat and raw milk from ill animals [2, 5, 12]. This is because of the greater inoculums from viremic animals such as sheep and cattle than that transmitted by mosquitoes thus providing an effective route for disease transmission [12, 31, 32].

In fact in a study conducted by [2] in Kenya, mosquito related exposures were not associated with severe RVF disease. Previous studies have suggested that inadequate information about disease transmission and prevention might contribute to adverse epidemiological effects including the spread of disease during an epidemic thus the need for proper public health information [26, 33]. It is therefore important to have an understanding of existing misconceptions about RVF because they may prevent people from taking protective action or from fully weighing their personal risk [19].

2. Engaging in certain dietary practices such as consumption of meat, raw milk and blood from diseased livestock does not cause RVF. Given that a majority of the community engaged in these dietary practices even during the RVF outbreaks and did not get sick, meant that RVF therefore was not transmitted through these practices. They associated RVF infection in humans with manifested symptoms and did not seem to know that majority of RVF human infections are asymptomatic [5].

In the Ijara region, meat, raw milk, ghee, blood and fat are the main benefits of livestock and form 80 % of the diet of the communities in this region [18]. This dependence on raw milk and blood from livestock predisposes the people to an RVF infection if the products are derived from an infected animal [18]. This study’s findings show that while the community had been informed of the risk factors associated with consumption of meat and milk from livestock during an RVF outbreak they did not believe it. They continued engaging in these practices with the perception that they would not be at risk since RVF was mainly caused by the mosquitoes. Indeed numerous studies have demonstrated that practices such as touching an aborted animal fetus, slaughtering, skinning and consumption of meat and milk from ill or dead livestock did play a key role in the transmission of RVF to humans [5, 32, 34].

Food preparation and consumption practices have already been identified as drivers of RVF transmission and spread in both animals and humans. Agro-pastoral communities in Tanzania identified consuming milk from sick animals and consumption of meat from dead animals as a transmission pathway for RVF [35]. In Tanzania as well, majority of the confirmed RVF cases in humans had a history of consumption of meat from dead sheep [8]. In the case of RVF, the beliefs about consumption of animal products during an RVF outbreak need to be addressed before the community can refrain from that practice. In their studies, [4, 12] also showed that the communities in Ijara District consumed meat from ill animals during the last outbreak to salvage the value of the protein of that animal.

In another study conducted in Saudi Arabia after an RVF outbreak, [36] found that there was a connection between RVF infection in humans and the consumption of raw milk in which concentrations of the RVF virus were found. This is in tandem also with previous studies conducted in the Ijara region which concluded that, the government’s ban on raw milk and home slaughter was difficult to enforce because livestock are critical to the livelihood of people in this region [2, 12, 14]. However, [26] noted in their study that the community was aware that infected domestic animals and aborted fetuses were entry risk pathways for RVF in Ijara district. Nevertheless, [26], observed that since they interviewed key informants exclusively they might have given their own expert opinion rather than the community’s perceptions. The need to have an understanding of existing misconceptions about RVF is great because they may prevent people from taking protective action or from fully weighing their personal risk [19].

3. Cooking meat that has been slaughtered from an ill animal makes it safe for consumption. The aspect of cooking the infected meat or boiling the milk may actually denature the virus but the highest risk is in slaughtering the sick animal, preparing the meat or milking, which may predispose the person performing these duties to be exposed to the secretions or aerosols of blood or body fluids that may cause RVF infection. Similarly in Sudan, the most dominant risk factor to cause RVF disease in humans was animal contact especially with aborted foetal material [7]. In the 2003 RVF outbreak in Egypt, it was found that RVF disease in humans occurred largely as a result of direct contact with animals during the slaughter of sick cattle [37].

Previous studies have shown that the aerosolization of blood and other body fluids during animal contact resulted to RVF infection for those exposed [2, 15, 29]. In their study, [2] noted that direct human contact with secretions from an animal infected with RVF contributed greatly to human RVF infections and concluded that certain exposures related to animal contact resulted to acute RVF infection. A previous study by [12] suggested that consumption of meat and milk from sick animals was the most significant risk factor for human infection with RVF. Similarly, in a study in Tanzania [29], a majority of the respondents reported that consuming milk from sick animals and meat from dead animals were risk factors for RVF. The perception that consuming cooked animal products, during an RVF outbreak, was safe needs to be addressed by stressing the fact that the greater risk lies in handling contaminated meat, without any protective barrier, either through slaughter, preparation or milking before cooking. Misconceptions limit people’s ability to change their behaviour [38] and hence the need to develop educational material that take into consideration the lay beliefs for use in continuous public health strategies.

### Strengths and limitations

This study was conducted 5 years after the last RVF outbreak of 2006/2007 hence, there could have been the challenge of recall bias. Nevertheless, RVF has a significant impact on the community thus the relevance of this study. In addition, the study utilized FGDs and narratives to get an insight of the lay perceptions of the community in relation to RVF. Although these participants were few and not a randomly selected sample, hence may not represent the whole community, they highlighted key issues related to their perceptions about risk factors for RVF in relation to their dietary practices and livelihood strategies that are worth exploiting since they influence effective control and management of the disease. The information they provided helps put into perspective the community dietary practices in relation to their beliefs about causation of RVF. However, further research should be conducted to establish the extent of these perceptions across the whole population and across different socio-demographic variables in the face of changing policy frameworks and socio-economic landscapes.

### Implications for control of Rift Valley Fever and other zoonoses

As per the health belief model [21], people’s beliefs about whether or not they are at risk of a certain disease and their perceptions of the benefits of taking action to avoid it influences their readiness to take action. The study findings have important implications for public health messaging for prevention and control interventions for RVF and other zoonoses.

Communities may hold lay beliefs about disease risk and this will influence their health promoting behavior either positively or negatively, hence the need to take this into consideration. For example, a government ban on slaughter and consumption of animal products did not deter the community in Ijara from slaughtering and consuming livestock products from sick animals. This ties in well with one of the precepts in the HBM which states that people take certain health related actions only if they believe that action will prevent a particular disease [21]. Their perceived susceptibility to RVF arising from their dietary practices in relation to livestock was nil. This is because they did not see these practices as risk factors for RVF given that this information did not tally with their lived experiences. People may also not perceive, interpret and act on risk information in the way expected based on various factors such as the extent to which individuals trust the information about disease risk, the source of that information, the channel used to convey the information, time taken to disseminate and how the information was disseminated. Using trusted sources and recognized channels and taking time to explain the risk factors to the communities taking into consideration their questions and concerns may help put the risk factors into perspective.

Dealing with risk factors that touch on people’s livelihoods may require a multi-pronged approach to provided alternatives for communities at risk especially in the face of a diseases outbreak. This may entail concerted efforts by relevant stakeholders to provide alternative means of food/livelihood during and immediately after the RVF outbreaks to mitigate against the challenges experienced by the community.

The community under study did not trust the RVF prevention messages that touched on their consumption of livestock products given that they did not fall ill from RVF. A key message that needs to be included in the public health interventions is the aspect of bio-safety when handling livestock products especially during an RVF outbreak. This needs to include using appropriate protective gear such as gloves, nose and mouth guards and observing basic sanitary measures such as hand washing and cleaning of the surfaces where the livestock products have been handled. This is because contact with direct secretions from an animal infected with RVF and or the aerosolization of blood and other body fluids during animal contact contributes greatly to RVF infection for those exposed [2, 15, 37]. The common and trusted channels and sources of information to the community also need to be assessed to enable timely delivery of public health messages in relation to RVF and through use of credible channels and sources. Communities may discount public health messages if their experiences prove otherwise. It is therefore crucial for RVF intervention programmes to develop detailed holistic messages that touch on all risk factors while interventions need to be accompanied by detailed explanations why they are being instituted and how they can help prevent transmission and spread of RVF. Successful initiatives have been noted, for example, effective health education campaigns during the 2006–2007 epizootic in Garissa supported by the local religious leaders (Imams) proved to be a critical step toward reducing human and animal morbidity and mortality caused by RVF alongside other measures like the ban on slaughter of animals and movement of livestock among others [12, 14]. A study on malaria in Malawi showed that a change in the health education given during ante natal clinics and distribution of sugar-coated chloroquine pills led to a 45 % increase in chloroquine utilization [39].

## Conclusion

The findings of this study show that while the community had heard, mainly from Government agencies, of the risks involved in consuming meat and milk during the RVF outbreaks, they did not believe that to be so. The main reason for this perception was that many of them reported to have consumed livestock products during the outbreaks of RVF yet they did not contract the disease. They also added that there were no alternative sources of food available during the RVF outbreak seasons as the roads were impassable and shops had been closed. In addition, the community highly value meat and milk from their livestock and they did not want to see that go to waste when their animals were dying in large numbers from RVF. The community also believes that once meat from a sick animal is cooked, it is free of any disease. They also believe that God protects them. These findings therefore imply that the perceived threat of risk of RVF infection from engaging in food consumption practices during an outbreak was low. Consequently, the willingness to adhere to preventive practices was minimal. In conclusion therefore the findings of this study show that the lay beliefs and perceptions of the community regarding the causes of RVF have implications on the community’s perceptions of risk and their willingness to engage in protective practices. Simply issuing food bans to deal with RVF is not sufficient. These need to be accompanied by comprehensive education and sensitization programs that include detailed explanations of the dynamics of RVF transmission to address any misconceptions arising from these interventions.

## Abbreviations

FGDs: focus group discussions
HBM: health belief model
KNBS: Kenya National Bureau of Statistics
RVF: Rift Valley fever
